# Activities Forgone because of Chronic Breathlessness: A Cross-Sectional Population Prevalence Study

**DOI:** 10.1089/pmr.2020.0083

**Published:** 2020-08-18

**Authors:** Slavica Kochovska, Sungwon Chang, Deidre D. Morgan, Diana Ferreira, Manraaj Sidhu, Rayan Saleh Moussa, Miriam J. Johnson, Magnus Ekström, David C. Currow

**Affiliations:** ^1^ IMPACCT, Faculty of Health, University of Technology Sydney, Ultimo, New South Wales, Australia.; ^2^ Australian national Palliative Care Clinical Studies Collaborative, Faculty of Health, University of Technology Sydney, Ultimo, New South Wales, Australia.; ^3^ Flinders University, Palliative and Supportive Services, RePaDD, Bedford Park, South Australia, Australia.; ^4^ Flinders University, Palliative and Supportive Services, Bedford Park, South Australia, Australia.; ^5^ Cancer Symptom Trials, Faculty of Health, University of Technology Sydney, Ultimo, New South Wales, Australia.; ^6^ Wolfson Palliative Care Research Centre, University of Hull, Hull, United Kingdom.; ^7^ Division of Respiratory Medicine & Allergology, Department of Clinical Sciences, Lund University, Lund, Sweden.

**Keywords:** breathlessness, dyspnea, symptom, symptom assessment

## Abstract

***Background:*** Chronic breathlessness is a prevalent disabling syndrome affecting many people for years. Identifying the impact of chronic breathlessness on people's activities in the general population is pivotal for designing symptom management strategies.

***Objective:*** This study aimed to evaluate the association between chronic breathlessness and activities respondents identify can no longer be undertaken (“activities forgone”).

***Design:*** This population-based cross-sectional online survey used a market research company's database of 30,000 registrants for each sex, generating the planned sample size—3000 adults reflecting Australia's 2016 Census by sex, age group, state of residence, and rurality.

***Setting/Subjects:*** The population of focus (*n* = 583) reported a modified Medical Research Council (mMRC) breathlessness scale ≥1 and experienced this breathlessness for ≥3 months.

***Measurements:*** Activities forgone were categorized by mMRC using coding derived from the Dyspnea Management Questionnaire domains. Activities were classified as “higher/lower intensity” using Human Energy Expenditure scale.

***Results:*** Respondents were male 50.3%; median age 50.0 (IQR 29.0); with 66% living in metropolitan areas; reporting 1749 activities forgone. For people with mMRC 1 (*n* = 533), 35% had not given up any activity, decreasing to 9% for mMRC 2 (*n* = 38) and 3% for mMRC 3–4 (*n* = 12). Intense sport (e.g., jogging and bike riding) was the top activity forgone: 42% (mMRC 1), 32% (mMRC 2), and 36% (mMRC 3–4). For respondents with mMRC 3–4, the next most prevalent activities forgone were “sexual activities” (14%), “lower intensity sports” (11%), and “other activities” (11%).

***Conclusions:*** People progressively reduce a wide range of activities because of their chronic breathlessness.

## Introduction

Chronic breathlessness is a disabling syndrome^1^ affecting >2.6% of the population daily, often for years.^2^ Function is compromised,^3^ creating a downward cycle of deconditioning,^4^ social isolation^5,6^ and physical dependence. Clinicians under-recognize the impact chronic breathlessness has on peoples' lives,^7,8^ often instead focusing on the underlying disease(s).^9^ Identifying the impact of chronic breathlessness on people's activities in the general population is pivotal for designing and promoting self-management and other symptom treatment strategies, and to help ultimately reduce unplanned health service utilization.^10,11^

The association between chronic breathlessness and impaired function has been demonstrated, but the range of the activities forgone as chronic breathlessness worsens has not been systematically captured at a population level. Describing any impact on populations (recruited independently of people's contact with health services) is important, given that chronic breathlessness remains largely invisible to health systems.^8,10^

This study aimed to evaluate the association between chronic breathlessness and the activities respondents identify can no longer be undertaken: activities of daily living and those activities that contribute to life's richness.

## Materials and Methods

This population-based cross-sectional cohort study used an online survey (November 2018). A market research company's database of up to 30,000 registrants for each sex generated the preplanned sample of 3000 responses representative of Australia's 2016 Census population^12^ by sex, 10 year age group, state/territory of residence, and rurality. Consenting adults (≥18 years) were eligible, and screened out if cells created using these four demographic domains were already filled by earlier respondents. No identifying information was collected. This community survey was governed by the Australian Market and Social Research Society code of conduct that aligns with best global industry standards for the conduct of social online research surveys and quantitative data collection. Consent from registrants was obtained at two separate time points: at the time of joining the database and at the time of participating in this particular survey.

The modified Medical Research Council (mMRC) breathlessness five-point ordinal scale^13^ assessed chronic breathlessness. Higher scores reflect less function before breathlessness limits exertion. Respondents with mMRC ≥1 were asked to nominate three activities they had given up because of their chronic breathlessness (“activities forgone”).

A coding frame combined responses to the “activities forgone” question with the Dyspnea Management Questionnaire (DMQ-30) “self-efficacy for activity” domain^14^ and the DMQ Computer Adaptive Test (DMQ-CAT) “activity avoidance” and “activity self-efficacy” domains.^15^ This is a validated measure for breathlessness in adults with chronic obstructive pulmonary disease (COPD). Classifying activities as “higher/lower intensity” (where applicable) followed Human Energy Expenditure scales.^16^

The population of particular interest for the analysis were defined by having an mMRC ≥1 for longer than three months duration (*n* = 583/3000). Activities forgone were categorized by mMRC scores. No data were imputed. The study is presented within the CHERRIES framework for online surveys.^17^

## Results

Study participants were male 50.3%; median age 50.0 (IQR 29.0); with 66% living in metropolitan areas (Table 1). They provided 1749 coded activities forgone (Table 2). Most people (*n* = 533; 91%) had mMRC = 1, nominating 1599 activities forgone.

**Table 1.** Domains Measured for Respondents to an Internet Survey on Breathlessness (*n* = 3000) Described by the Modified Medical Research Council Breathlessness Scale

	Breathlessness (mMRC 1–4) and ≥3 months duration (n = 583)			
Domain measured	mMRC 1 (n = 533)	mMRC 2 (n = 38)	mMRC 3–4 (n = 12)	Total (n = 583)
**Age**				
Mean (SD)	50.6 (15.9)	46.8 (16.0)	46.6 (20.6)	50.3 (16.1)
Med (IQR)	51.0 (28.0)	44.5 (31.0)	40.5 (43.0)	50.0 (29.0)
18–34	115 (21.6)	11 (28.9)	5 (41.7)	131 (22.5)
35–44	99 (18.6)	8 (21.1)	1 (8.3)	108 (18.5)
45–54	85 (15.9)	8 (21.1)	1 (8.3)	94 (16.1)
55–64	99 (18.6)	2 (5.3)	1 (8.3)	102 (17.5)
65 and older	135 (25.3)	9 (23.7)	4 (33.3)	148 (25.4)
Female	268 (50.3)	19 (50)	3 (25)	290 (49.7)
Male	265 (49.7)	19 (50)	9 (75)	293 (50.3)
Greater capital city	355 (66.6)	21 (55.3)	7 (58.3)	383 (65.7)
Rest of state/territory	160 (30)	16 (42.1)	5 (41.7)	181 (31)
No response	18 (3.4)	1 (2.6)	0 (0)	19 (3.3)
Current smoker	33 (6.2)	3 (7.9)	2 (16.7)	38 (6.5)
Former smoker	160 (30)	12 (31.6)	5 (41.7)	177 (30.4)
Never smoked	293 (55)	21 (55.3)	4 (33.3)	318 (54.5)
Prefer not to say	2 (0.4)	0 (0)	1 (8.3)	3 (0.5)
No response	45 (8.4)	2 (5.3)	0 (0)	47 (8.1)
No anxiety or depression	418 (78.4)	22 (57.9)	8 (66.7)	448 (76.8)
Anxiety only	20 (3.8)	4 (10.5)	0 (0)	24 (4.1)
Depression only	37 (6.9)	3 (7.9)	2 (16.7)	42 (7.2)
Anxiety and depression	56 (10.5)	9 (23.7)	1 (8.3)	66 (11.3)
No response	2 (0.4)	0 (0)	1 (8.3)	3 (0.5)
AKPS 100–70	520 (97.6)	32 (84.2)	10 (83.3)	562 (96.4)
AKPS ≤60	12 (2.3)	6 (15.8)	1 (8.3)	19 (3.3)
Unable to determine	1 (0.2)	0 (0)	1 (8.3)	2 (0.3)

AKPS, Australia-modified Karnofsky Performance Scale; mMRC, modified Medical Research Council.

**Table 2.** Activities Forgone Nominated by Respondents with Self-Reported Chronic Breathlessness (mMRC ≥1 for ≥3 Months) in a General Population Sample (*n* = 583)

Activities forgone	Chronic breathlessness (mMRC ≥1 and ≥3 months duration) (n = 583)			
	Total nominated responses (n = 1749)^a^	mMRC 1 (n = 1599)^a^	mMRC 2 (n = 114)^a^	mMRC 3–4 (n = 36)^a^
No trouble^b^	575 (33%)	564 (35%)	10 (9%)	1 (3%)
Sports (higher intensity)	715 (41%)	665 (42%)	37 (32%)	13 (36%)
Sexual activities	40 (2%)	30 (2%)	5 (4%)	5 (14%)
Sports (lower intensity)	57 (3%)	36 (2%)	17 (15%)	4 (11%)
Other	98 (6%)	83 (5%)	11 (10%)	4 (11%)
Gardening/doing yard work	68 (4%)	57 (4%)	8 (7%)	3 (8%)
Strenuous/physical everyday activities	39 (2%)	32 (2%)	5 (4%)	2 (6%)
Hobbies	20 (1%)	14 (1%)	4 (4%)	2 (6%)
Household chores	47 (3%)	39 (2%)	7 (6%)	1 (3%)
Work (lower intensity)	24 (1%)	18 (1%)	5 (4%)	1 (3%)
Caregiving responsibilities for other people/pets	23 (1%)	21 (1%)	2 (2%)	0 (0%)
Shopping (e.g., groceries)	13 (1%)	11 (1%)	2 (2%)	0 (0%)
Visiting friends or family in their home (going out, socializing)	6 (0%)	5 (0%)	1 (1%)	0 (0%)
Work (higher intensity)	19 (1%)	19 (1%)	0 (0%)	0 (0%)
Driving	5 (0%)	5 (0%)	0 (0%)	0 (0%)
Personal care (e.g., getting dressed, showering, and bathing)	0 (0%)	0 (0%)	0 (0%)	0 (0%)
Traveling/holidays	0 (0%)	0 (0%)	0 (0%)	0 (0%)

^a^ Respondents (*n* = 583) were able to nominate up to three activities that have been impacted by their breathlessness in response to the question “Please describe the three most important things that you have had to give up because of your shortness of breath.”

^b^ Nominated responses include Nothing/Hasn't given up anything but has slowed down or dealt with it/Don't know/No response.

For people with mMRC 1 (*n* = 533), 35% of responses indicated that they had not given up any activity, decreasing to 9% for people with mMRC 2 (*n* = 38) and 3% for people with mMRC 3–4 (*n* = 12; Table 2). Intense sport (e.g., running, jogging, and bike riding) was the top activity forgone indicated in 42% (mMRC 1), 32% (mMRC 2), and 36% (mMRC 3–4) of responses. This was followed by “other activities” (e.g., volunteering and getting into the car) (5%) and “gardening/doing yard work” (4%) for mMRC 1; and “lower intensity sports” (15%) and “other activities” (10%) for mMRC 2. For respondents with mMRC 3–4, the second most nominated activity forgone was “sexual activities” (14%), followed by “lower intensity sports” (11%) and “other activities” (11%).

## Discussion

This population-based study of people with chronic breathlessness (most of whom would attribute their breathlessness to a respiratory cause)^18^ paints a picture of progressive limitations in activities as the syndrome worsens, with more strenuous activities first affected. At every level of worsening chronic breathlessness, activities of daily living become more difficult as do household chores such as gardening. The magnitude of change is consistent with previous findings showing that >50% of community-dwelling people aged ≥70 years live with breathlessness that is severe enough to restrict their activities^3^ and, sadly, contribute to mortality predictions.^19^

Hobbies and recreational activities seem to be most commonly affected by chronic breathlessness. Viewed in the context of Maslow's hierarchy of needs,^20^ people give up higher tier activities early, but this progresses for many people to basics of day-to-day existence, including household chores. Breathlessness impinges on the ability to perform activities outside of the home earlier than in-home activities,^21^ reflecting a shrinking LifeSpace^22^ and increasing social isolation, with potential implications for caregivers and changing roles within the household. Given the known relationship between social isolation and psychological morbidity (also associated with chronic breathlessness),^23^ this may be a target for an intervention such as social prescribing.^24^

Sexual activity diminishes for people with most severe chronic breathlessness (mMRC 3–4). This is consistent with results from a population study that has shown an association between chronic breathlessness and the prevalence and duration of sexual inactivity in people aged ≥65 years.^25^ Sexual function and sexual well-being are important aspects of personhood across the age spectrum, including for people with chronic progressive illnesses and life-limiting illnesses.^26–28^ A better understanding of the sexual health of people with chronic breathlessness (and their partners) could help clinicians to proactively help people to optimize their sexual activities.^3^ Eliciting this impact in clinical consultations is critical in ensuring patient-centered communication.^29–32^

Actively avoiding things that cause breathlessness may lead patients to underestimate the severity of their chronic breathlessness.^7^ Developing a conversation around the impact of the symptom will help to redress known under-reporting by patients and caregivers.^33^ Although not explicitly asked in this study, responses indicated that people may be reducing (rather than stopping) their daily activities or not enjoying participating in such activities because of their chronic breathlessness.

A limitation is the cross-sectional design that evaluates associations but limits causal inference. The impact of chronic breathlessness on activities was self-reported, with no objective verification. By sampling against key demographic parameters to reflect directly the population seen in the national census (and, therefore, not limited by case finding through having contact with health services), the findings are likely to be generalizable to the whole population. The quota sampling method enables replication for other populations by setting identical parameters. The online delivery of the survey, however, may have introduced bias toward participants who are more technologically adept, educated, or from higher socioeconomic strata, thus limiting the generalizability to the population as a whole.

The present findings have several implications. Current history taking for people with chronic conditions often fails to identify and assess the impact of the symptom,^34^ partly due to inadequate clinical enquiry, and partly due to under-reporting by people who have reduced or forgone activities often as that would have been part of their lives previously. This highlights the need for a more effective and systematic assessment of chronic breathlessness, including its presence, severity, and impact on everyday lives. Timely and accurate identification of the impact of the symptom, and routine evaluation over the course of the illness, will facilitate more targeted self-management options for people who live with this symptom.^11,21,35^ At the health system level, systematic screening and assessment may help to reduce the unplanned use of primary and secondary health care, including acute-on-chronic breathlessness presentations to emergency departments and hospital admissions.^10,36–39^

Future therapeutic interventions should aim to improve people's ability to perform activities both for basic self-care, and to enrich life, reduce/prevent social isolation, and its impact on mental well-being; and to stop and/or reverse deconditioning.^40,41^ Allied health such as occupational therapists and physiotherapists play a key role in optimizing function in this setting.

The impact on caregivers should not be underestimated. As people reduce or cease activities, caregivers will be required to provide increasing physical and psychological care. Equally, this raises questions about providing services for people who may not have a caregiver as they live with an increasing functional impairment due to chronic breathlessness.

## Abbreviations Used

DMQ: Dyspnea Management Questionnaire
DMQ-CAT: Dyspnea Management Questionnaire Computer Adaptive Test
mMRC: modified Medical Research Council
